# USP14 deficiency inhibits neointima formation following vascular injury via degradation of Skp2 protein

**DOI:** 10.1038/s41420-024-02069-1

**Published:** 2024-06-22

**Authors:** Xiaohong Xia, Xiaolin Liu, Qiong Xu, Jielei Gu, Sisi Ling, Yajing Liu, Rongxue Li, Min Zou, Siqin Jiang, Zhiwei Gao, Canshan Chen, Shiming Liu, Ningning Liu

**Affiliations:** 1 Department of Cardiology, Guangzhou Institute of Cardiovascular Disease, Guangdong Key Laboratory of Vascular Diseases, State Key Laboratory of Respiratory Disease, the Second Affiliated Hospital, Guangzhou Medical University, Guangzhou, 510260 China; 2 Affiliated Cancer Hospital & Institute of Guangzhou Medical University, Guangzhou Municipal and Guangdong Provincial Key Laboratory of Protein Modification and Degradation, School of Basic Medical Sciences, Guangzhou Medical University, Guangzhou, 510095 China

**Keywords:** Restenosis, Deubiquitylating enzymes

## Abstract

Ubiquitin-proteasome system (UPS) is involved in vascular smooth muscle cell (VSMC) proliferation. Deubiquitinating enzymes (DUBs) have an essential role in the UPS-regulated stability of the substrate; however, the function of DUBs in intimal hyperplasia remains unclear. We screened DUBs to identify a protein responsible for regulating VSMC proliferation and identified USP14 protein that mediates cancer development, inflammation, and foam cell formation. USP14 promotes human aortic smooth muscle cell and A7r5 cell growth in vitro, and its inhibition or deficiency decreases the intimal area in the mice carotid artery ligation model. In addition, USP14 stabilizes Skp2 expression by decreasing its degradation, while Skp2 overexpression rescues USP14 loss-induced issues. The current findings suggested an essential role of USP14 in the pathology of vascular remodeling, deeming it a promising target for arterial restenosis therapy.

## Introduction

Atherogenesis is the major cause of cardiovascular diseases, which are the leading causes of death in developed countries [1, 2]. Patients with coronary atherosclerosis require indispensable surgical procedures, such as stenting, angioplasty, bypass surgery, and atherectomy. Surgical procedures are required for mechanical injuries to the arterial vascular system. Vascular restenosis occurs after surgical procedures that limit the prognosis of the patients [3]. Several studies have shown that arterial intimal hyperplasia is a pathological issue of restenosis. Vascular smooth muscle cells (VSMCs) migrate, proliferate, differentiate, and secrete extracellular matrix, which are the leading pathophysiological processes underlying neointima formation [4]. The prominent pathological event of VSMC proliferation spans the media to the intima; it does not occur in normal adult arteries but rather in tissues with failed transplant arteriosclerosis and venous grafts [5]. Therefore, VSMC proliferation regulation is critical for reducing intimal hyperplasia.

VSMC proliferation is mediated by a myriad of factors. Among these factors, cell cycle regulation is a promising strategy for the treatment of intimal hyperplasia disease. Mechanical injury induces VSMC proliferation by promoting the G1 to S phase transition [6]. The inactivation and phosphorylation of retinoblastoma (Rb) plays a critical role in the G1 phase of cell cycle progression, trigged by A, D, and E cyclins and the associated cyclin-dependent kinases (CDK4, CDK2, and CDK6). Moreover, the cyclin complexes are negatively mediated by the cyclin-dependent kinase inhibitors (CDKIs), such as p21, p27, p16, and p57. CDKIs are downregulated in the event of mechanical injury in vivo, followed by the activation of cyclin-related proteins that induce G1/S phase transition [7, 8]. p27 is a key protein of the CDKI family [9]. The deletion of p27 induces a vascular phenotype characterized by increasing neointima formation in the event of arterial injury [10]. Moreover, p27 is a downstream substrate regulated by S-phase kinase-associated protein 2 (Skp2), a ubiquitin ligase, promoting the polyubiquitination and proteasomal degradation of p27 [11–13]. Recent studies have demonstrated that decreasing Skp2 expression inhibits VSMC proliferation in vitro and in vivo, thereby affecting the final neointimal thickening [14–16]. Thus, regulating Skp2 protein is crucial for VSMC plasticity.

Protein turnover depends on the ubiquitin-proteasome system (UPS) that is involved in the protein degradation pathways associated with cell cycle, apoptosis, signaling, and differentiation. Importantly, inhibition of proteasome or ubiquitin ligases decreases VSMC proliferation and apoptosis to prevent neointima formation [17–19]. UPS consists of E1, E2, E3, and deubiquitinases (DUBs). Some of these members have been investigated in VSMC proliferation. For example, carboxyl terminus of Hsc70-interacting protein (CHIP) is an E3 ligase that increases VSMC proliferation via p27 and FoxO1 degradation [20, 21]. Nonetheless, whether DUBs are associated with VSMC proliferation has not yet been investigated. Previously, we observed that ubiquitin Specific Peptidase 10 (USP10) promotes neointima formation by deubiquitination and stabilization of Skp2 [22]. Thus, in this study, we sought to explore the role of USP14 during neointimal thickening.

## Results

### Growth factor stimuli or carotid artery ligation upregulates USP14

**Fig. 1:**
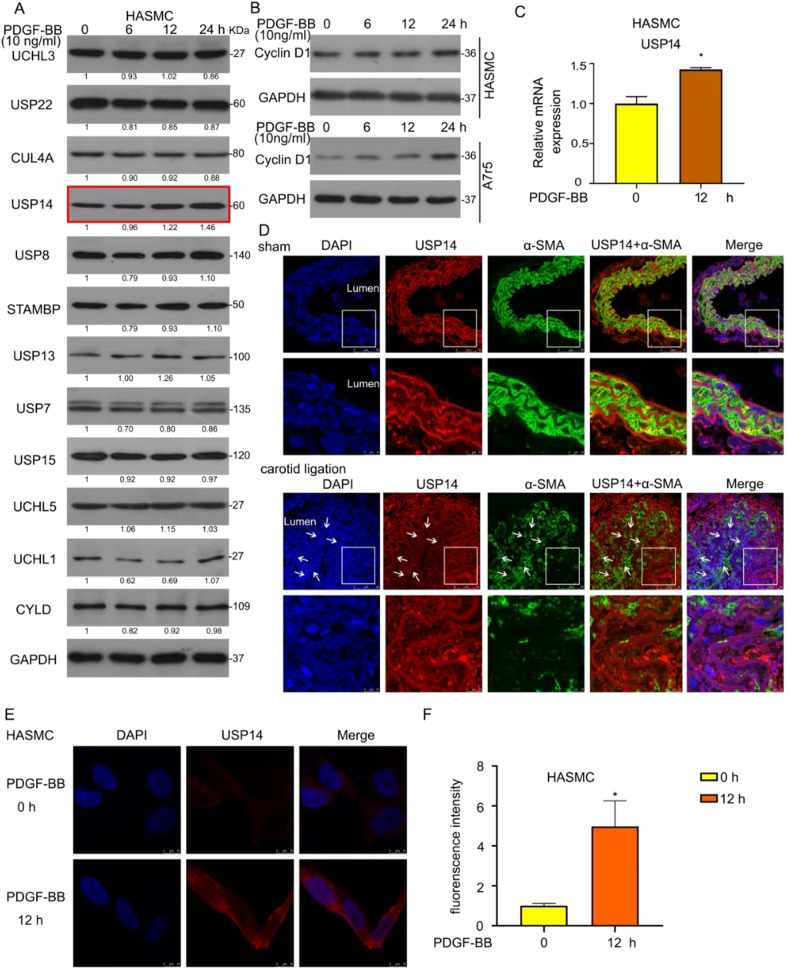
Growth factor stimuli or carotid artery ligation upregulates USP14. **A**, **B** Cells were starved with serum-free medium for 24 h. Protein lysate was obtained from HASMCs treated with PDGF-BB (10 ng/mL), followed by Western blot. GAPDH was a loading control. **C** Total RNA was extracted from HASMCs exposed to PDGF-BB. RT-qPCR was applied to evaluate the mRNA level of *USP14* (*n* = 3). **D** Immunofluorescence staining with USP14 (red), α-SMA (green), and DAPI (blue) on the carotid arteries of ligation and sham groups. **E** Immunofluorescence staining with USP14 in PDGF-BB-treated HASMCs for 12 h. **F** Fluorescence intensity was measured using Image J (n = 3). *P < 0.05.

UPS is closely related to VSMC proliferation; however, the role of DUBs in this progression is yet unknown. Therefore, we measured the expression of several proteins under the treatment of PDGF-BB, a key mediator in vascular injury, and potent stimuli of VSMCs for different periods. The results of Western blot assay showed that USP14 protein expression was upregulated among a series of DUBs after PDGF-BB stimuli in a time-dependent manner (Fig. 1A). Next, we tested the effect of PDGF-BB on VSMC proliferation and found that Cyclin D1 level was upregulated, indicating that PDGF-BB promotes VSMC proliferation (Fig. 1B). In addition to protein expression, we evaluated the mRNA level of *USP14*. RT-qPCR results showed that PDGF-BB upregulates the mRNA level of *USP14* in human aortic smooth muscle cells (HASMCs) (Fig. 1C). Furthermore, we found ubiquitous expression of USP14 in a vascular injury model of complete carotid ligation using immunofluorescence staining assay. Notably, USP14 expression was higher compared to the sham group after ligation, suggesting that the upregulation of the protein was triggered during intimal hyperplasia (Fig. 1D). Moreover, the fluorescence intensity of USP14 was strong after 12 h of PDGF-BB treatment in HASMCs (Fig. 1E, F).

### Inhibition or knockdown of USP14 suppresses VSMC growth in vitro

**Fig. 2:**
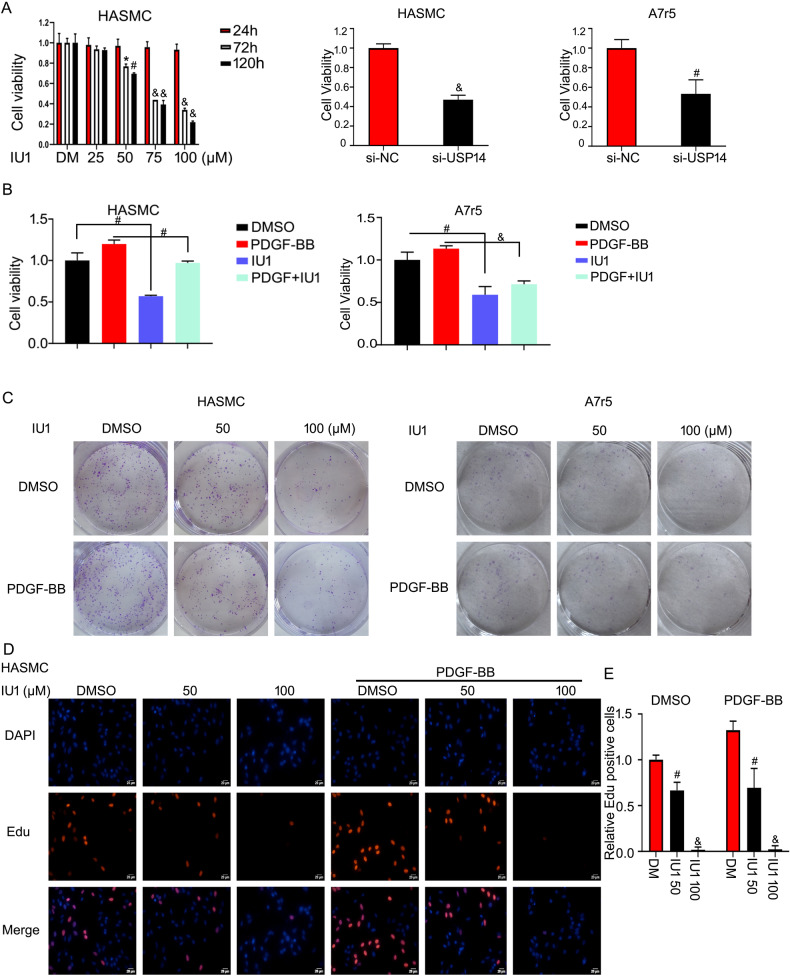
Inhibition or knockdown of USP14 suppresses VSMC growth in vitro. HASMCs were starved for 24 h. **A** HASMCs were treated with IU1 (0, 25, 50, 75, and 100 μM) for 24, 48, and 72 h. MTS agent was added to test cell viability, and VSMCs were treated with USP14 siRNA for 48 h, followed by MTS assay (*n* = 3). **B** Starved HASMCs and A7r5 cells were treated with IU1 and/or PDGF-BB (*n* = 3). **C** The treated cells were cultured for 10–14 days and stained with crystal violet. **D** Cells were exposed to IU1 and/or PDGF-BB for 48 h and subjected to EdU staining. **E** Number of stained cells. Data are presented as mean ± SD from three independent experiments (*n* = 3). *P < 0.05, ^#^*P* < 0.01, ^&^*P* < 0.001.

Cell proliferation is the leading pathophysiological basis of intimal hyperplasia. Since USP14 is upregulated during neointima formation, we tested whether it plays a role in VSMC proliferation based on cell viability. The results showed that USP14 inhibitor (IU1) inhibits HASMC growth. The off-target effects of the inhibitor were circumvented using small interfering RNA (siRNA) USP14; the results showed that USP14 knockdown suppresses cell proliferation in HASMCs and A7r5 cells (Fig. 2A). PDGF-BB triggers cell proliferation and is a key factor on intimal hyperplasia. We also found that IU1 inhibits VSMC growth under PDGF-BB stimuli (Fig. 2B). The prolonged antiproliferative effect of USP14 deletion was assessed by colony formation assay, and the results showed that IU1 inhibits the formation of cell colonies (Fig. 2C). EdU staining assay for measuring DNA replication showed that IU1 concentration-dependently decreased the number of stained cells in the presence and absence of PDGF-BB stimuli (Fig. 2D, E).

### Inhibition or knockdown of USP14 suppresses cell cycle transition and migration

**Fig. 3:**
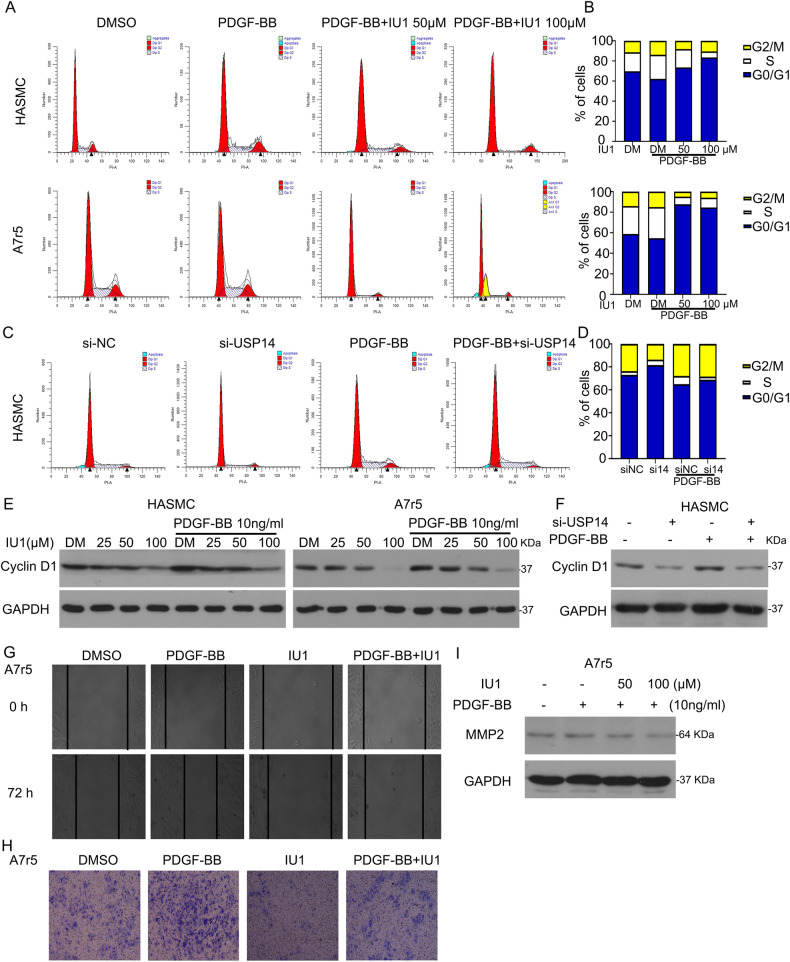
Inhibition or knockdown of USP14 suppresses cell cycle transition and migration. **A** Cell distribution was assessed on VSMCs treated with PDGF-BB or/and IU1 (75 μM) using flow cytometry. **C** HASMCs were treated with PDGF-BB or/and USP14 siRNA for 48 h, followed by flow cytometry. **B**, **D** Cell number was counted at different phases. **E** Cells were starved for 24 h and exposed to IU1 and/or PDGF-BB for 48 h. Western blot was used to evaluate the expression of Cyclin D1. **F** HASMCs were treated with USP14 siRNA and/or PDGF-BB for 48 h, followed by Western blot. **G** A7r5 cells were exposed to PDGF-BB and/or IU1, followed by scratch assay. Images were captured at 0 h and 72 h. **H** The treated cells were transferred to the transwell for migration. **I** Protein extracts were collected for Western blot to evaluate MMP2 expression.

VSMC proliferation depends on G1 to S phase transition of the cell cycle; hence, we speculated that USP14 promotes cell cycle progression. This hypothesis was tested using flow cytometry to detect cell distribution. Indubitably, cell cycle arrest at G0/G1 phase was induced by USP14 inhibition (Fig. 3A, B). Moreover, USP14 knockdown blocked the G1 to S phase transition (Fig. 3C, D). The proteins associated with cell cycle progression at G0/G1 phase were evaluated by Western blotting. USP14 inhibition or knockdown downregulated the G0/G1 phase protein, Cyclin D1 (Fig. 3E, F). In addition to cell cycle, cell migration also causes intima thickening. Scratch assay showed that IU1 slows down wound healing in A7r5 cells (Fig. 3G). Transwell assay revealed that there were fewer migratory cells in the of USP14 inhibitor-treated group than in the control group, suggesting that USP14 inhibition impairs cell migration (Fig. 3H). Western blot assay showed that IU1 decreases the expression of MMP2 protein (Fig. 3I).

### USP14 mediates the expression of Skp2 protein

**Fig. 4:**
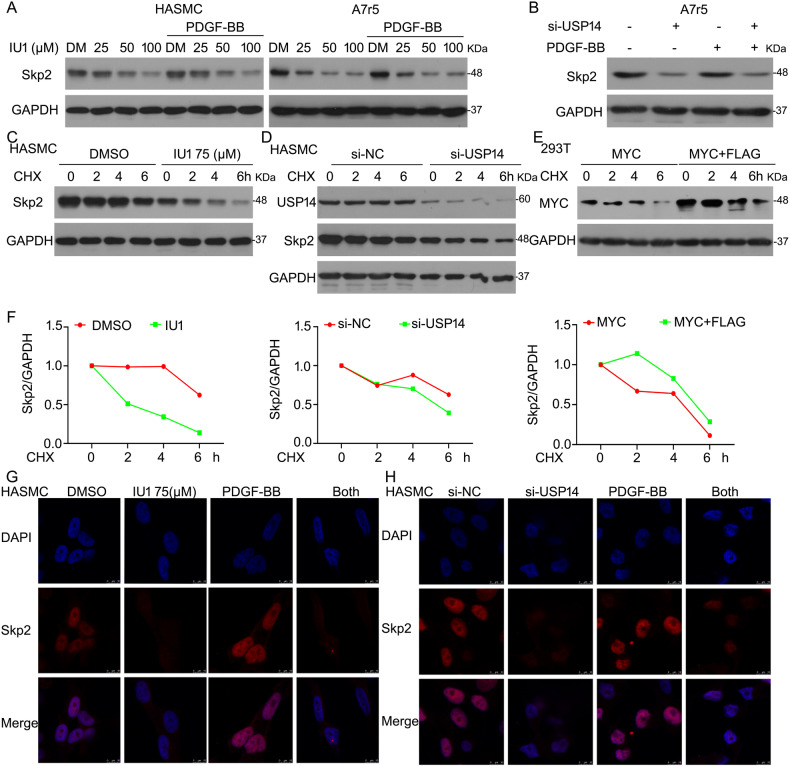
USP14 mediates the expression of Skp2 protein. **A** VSMCs were exposed to IU1 and/or PDGF-BB, followed by Western blot assay for Skp2 expression. **B** A7r5 cells were transfected with USP14 siRNA and/or PDGF-BB. Western blot assay was conducted to detect Skp2 expression. **C**, **D** HASMCs were treated with CHX and IU1/USP14 siRNA+CHX for 2, 4, and 6 h, respectively. Western blot assay was performed to assess Skp2 protein. **E** HEK293T cells were transfected with MYC-Skp2 or MYC-Skp2+FLAG-USP14 for 48 h and treated with CHX, followed by western blot assay. **F** The intensity of Skp2 band was quantified by Image J. **G**, **H** HASMCs were treated with IU1/USP14 siRNA and/or PDGF-BB. Immunofluorescence staining with USP14 was assessed by confocal microscopy.

*Skp2* is the primary gene promoting VSMC proliferation [16] and has been considered a target for controlling cell proliferation [23]. Next, we investigated whether USP14-mediated Skp2 protein expression affects cell proliferation. Firstly, we used Western blot assay and found that USP14 inhibitor downregulates Skp2 expression. Also, Skp2 expression increased with PDGF-BB stimulation, whereas IU1 showed a reverse phenomenon in HASMC and A7r5 cells (Fig. 4A). Additionally, USP14 siRNA induced-Skp2 expression in the absence and presence of PDGF-BB, as assessed by western blot assay (Fig. 4B). To explore the mechanism of Skp2 downregulation by USP14 silencing, we applied cycloheximide (CHX) to block protein synthesis. The results showed that inhibition or knockdown of USP14 accelerates the downregulation of Skp2 protein, indicating that USP14 deletion promotes the degradation of Skp2 (Fig. 4C, D, F). To further confirm this effect, we transfected HEK293T cells with MYC-tagged Skp2 and FLAG-tagged USP14 plasmids. Western blot results showed that the overexpression of USP14 partially prevents the degradation of Skp2 protein (Fig. 4E, F). Next, we observed that USP14 inhibitor or siRNA decreases the fluorescence intensity of Skp2 but does not alter its translocation, according to immunofluorescence staining (Fig. 4G, H).

### Interaction between Skp2 and USP14

**Fig. 5:**
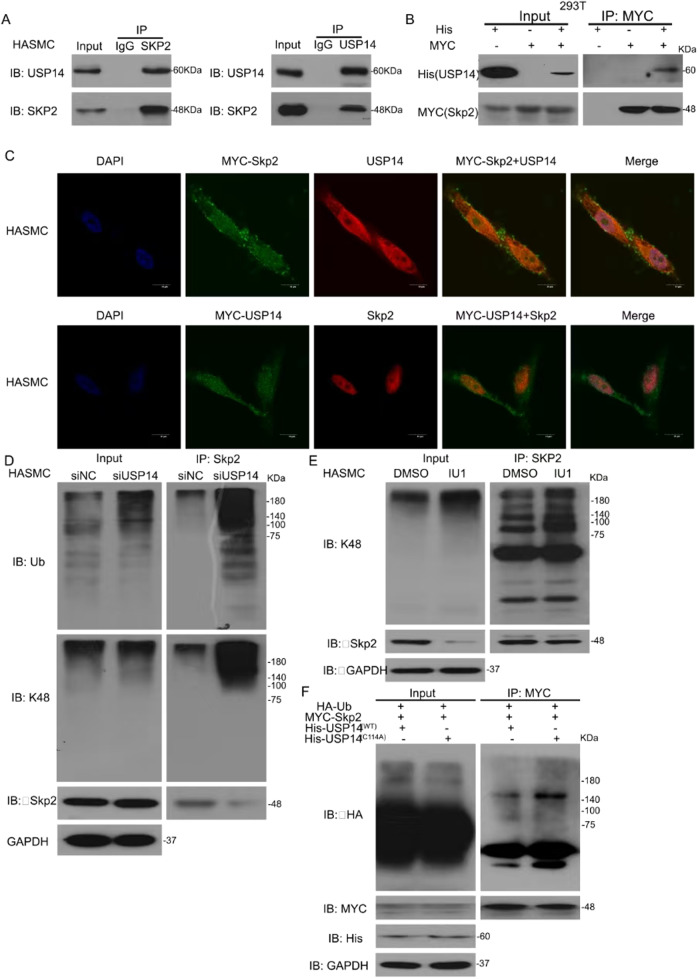
Interaction between Skp2 and USP14. **A** Protein lysate was obtained from HASMCs. IP with anti-Skp2 antibody or anti-USP14 antibody, immunoblot with USP14 and Skp2. **B** HEK293T cells were transfected with His-tagged USP14 and MYC-tagged Skp2. IP with MYC, IB with His and MYC. **C** HASMCs transfected with MYC-tagged Skp2/MYC-USP14 were stained with anti-MYC and anti-USP14/Skp2 antibodies, followed by confocal microscopy. **D** HASMCs were exposed to USP14 siRNA for 48 h, followed by IP with Skp2 and IB for Ub and K48 proteins. **E** HASMCs were treated with IU1. IP with Skp2 and IB with K48. **F** HEK293T cells were transfected with HA-Ub, His-USP14 (WT), His-USP14 (C114A), and Myc-Skp2. IP with MYC and IB with HA.

DUBs prevent the degradation of their substrate through protein-protein interaction and posttranslation. In this study, we found that USP14 loss suppresses Skp2 expression. To explore the underlying molecular mechanism, we used a co-immunoprecipitation (Co-IP) assay to assess the interaction between Skp2 and USP14 proteins and observed that Skp2 significantly interacted with endogenous USP14 and vice versa (Fig. 5A). Cellular proteins were extracted from HEK293T cells transfected with His-tagged USP14 and Myc-tagged Skp2 and analyzed by Co-IP and Western blot assays. The results showed that exogenic His-tagged USP14 was bound to Myc-tagged Skp2, indicating a strong interaction between USP14 and Skp2 proteins (Fig. 5B). Next, we tested the target protein localization and expression by immunofluorescence staining and confocal microscopy and found that Myc-tagged Skp2 was colocalized with endogenous USP14 in HASMCs; also, MYC-tagged USP14 was colocalized with Skp2 (Fig. 5C). Protein–protein interaction assay assessed the posttranslational modification of Skp2. The expression of poly- and K48-ubiquitinated Skp2 was increased by USP14 knockdown (Fig. 5D). Consistently, USP14 inhibition dramatically upregulated K48-ubiquitinated Skp2 level (Fig. 5E). HEK293T cells expressing His-tagged USP14 (USP14/WT) and His-tagged catalytically inactive mutant of USP14 (USP14/C114A) were prepared. Co-IP and Western blot analysis showed that the polyubiquitination of Skp2 was increased in HEK293T cells expressing USP14/C114A (Fig. 5F). These results indicated that USP14 interacts with Skp2 and reduces the degradation of Skp2 protein.

### Role of USP14 and Skp2 in VSMC proliferation

USP14 inhibition or knockdown suppressed cell proliferation and blocked G1 to S phase transition in HASMCs and A7r5 cell cycle. In order to confirm the effect of USP14 on VSMCs, we evaluated the cell proliferation of VASMCs overexpressing USP14. The cell viability was increased in HASMCs and A7r5 cells transfected with MYC-tagged USP14 (Fig. S1A). Western blot assay showed an increased expression of USP14 protein, indicating the overexpression of USP14. Furthermore, proteins related to the cell cycle were regulated as follows. Cyclin D1 was upregulated, and p27 was downregulated. Importantly, the expression of USP14 target protein, Skp2, was increased (Fig. S1B). Cell cycle distribution at G0/G1 was less in the USP14 group than in the control group (Fig. S1C, D), indicating that USP14 promotes cell cycle progression from the G1 to the S phase. Although Skp2 plays a critical role in VSMC proliferation, we examined the function of Skp2 and found that its overexpression increases cell viability, while the expression of the downstream p27 was decreased (Fig. S1E, F); also, similar to USP14, the cell cycle transition was promoted by Skp2 (Fig. S1G, H).

### USP14 promotes VSMC proliferation in a Skp2-dependent manner

**Fig. 6:**
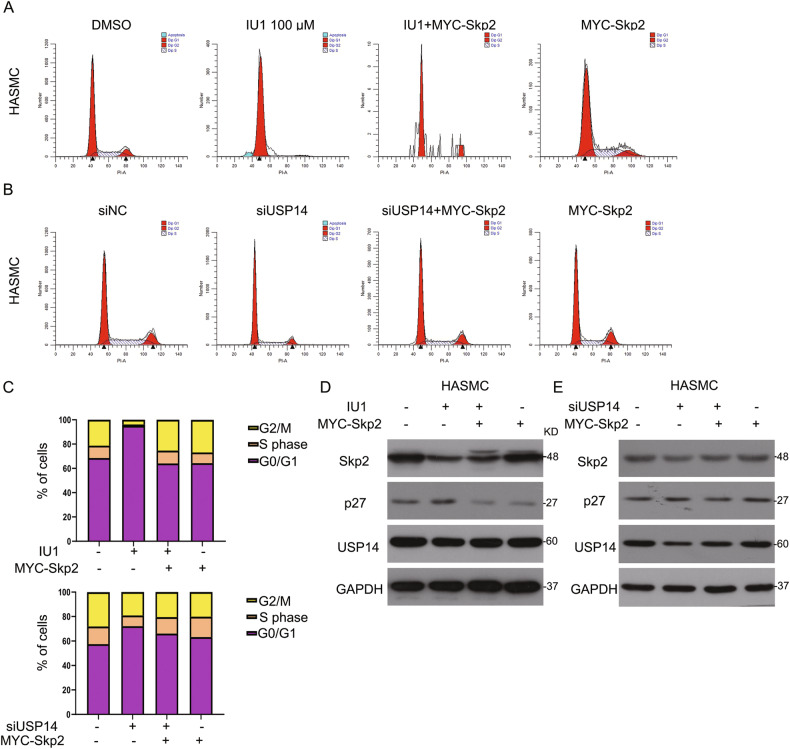
USP14 promotes VSMC proliferation in a Skp2-dependent manner. **A**, **B** Cells were treated with IU1/USP14 siRNA or/and PDGF-BB, followed by flow cytometry. **C** Cell number at various phases. **D**, **E** Western blot assay was performed to measure the expression of Skp2, p27, and USP14. GAPDH was used as the loading control.

Based on the above findings, we speculated whether USP14 regulates cell growth via stabilizing Skp2 protein. To test this hypothesis, HASMCs were transfected with the full-length plasmid of human Skp2, and the function of the protein on USP14 inhibition-induced cell cycle arrest was assessed. As shown in Fig. 6A, cell growth inhibition triggered by IU1 was rescued by Skp2 overexpression. Skp2 also attenuated G1 to S phase arrest induced by USP14 siRNA (Fig. 6B, C). Next, we used Western blot assay to evaluate the expression of some cycle proteins. Notably, IU1 induced-Skp2 downregulation and increased p27, which blocks G0/G1 to S phase progression, was suppressed by Skp2 plasmid (Fig. 6D). Similar results were observed when HASMCs were transfected with USP14 siRNA and Skp2 plasmid (Fig. 6E).

### USP14 deficiency attenuates neointima formation in vivo

**Fig. 7:**
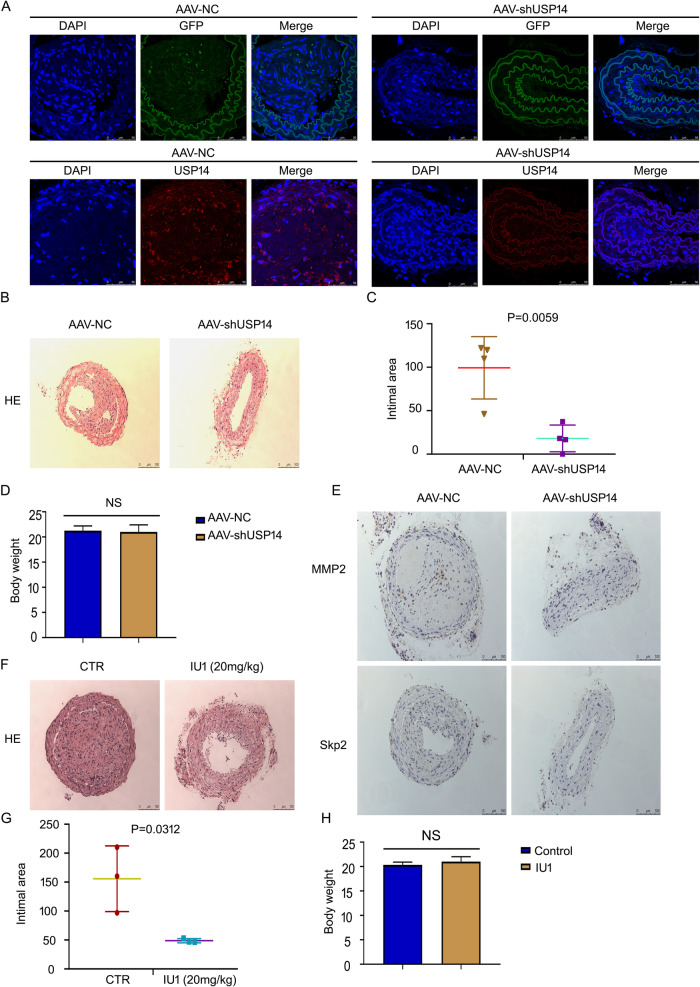
USP14 deficiency attenuates neointima formation in vivo. **A** Immunofluorescence staining demonstrates GFP (green) and USP14 (red) expression in the ligated carotid of mice. **B** Neointima formation in the carotid arteries between the control and AAV-USP14 shRNA groups using HE staining. **C** Quantified intimal areas (n = 4 after 21 days). **D** Body weight (g) after 21 days (n = 4). **E** Skp2 and MMP9 expression in the ligated carotid using IHC. **F** HE-stained carotid is shown in the mice model of ligation after 21 days in the control and IU1 groups. **G** Intimal area and **H** body weight (g) (n = 3).

Since USP14 was upregulated in VSMCs under PDGF-BB stimuli, we hypothesized that USP14 contributes to restenosis in SMCs after injury. This hypothesis was investigated using USP14 shRNA-transfected mice. Firstly, we confirmed the knockdown effect of GFP-tagged AAV-USP14 shRNA. Immunofluorescence showed that GFP (green) was expressed in carotid, and the expression of USP14 was attenuated in AAV-USP14 shRNA compared to the control group, suggesting successful USP14 deletion in mice (Fig. 7A). USP14 deficiency significantly decreased neointimal formation, as reflected by intima areas, further indicating that vascular hyperplasia was inhibited by USP14 loss (Fig. 7B, C). We also assessed the condition of mice based on body weight, and no difference was detected between the two groups (Fig. 7D). Skp2 and MMP2 associated with SMC proliferation and migration from media to intima were evaluated using immunohistochemistry (IHC) assay (Fig. 7E); USP14 silencing attenuated the expression of the proteins. To further demonstrate the effect of USP14, mice were treated with IU1; the intima area was decreased by IU1 (Fig. 7F, G), whereas no effect was detected on the body weight (Fig. 7H). These results indicated that USP14 has a driving role in SMC proliferation.

## Discussion

**Fig. 8:**
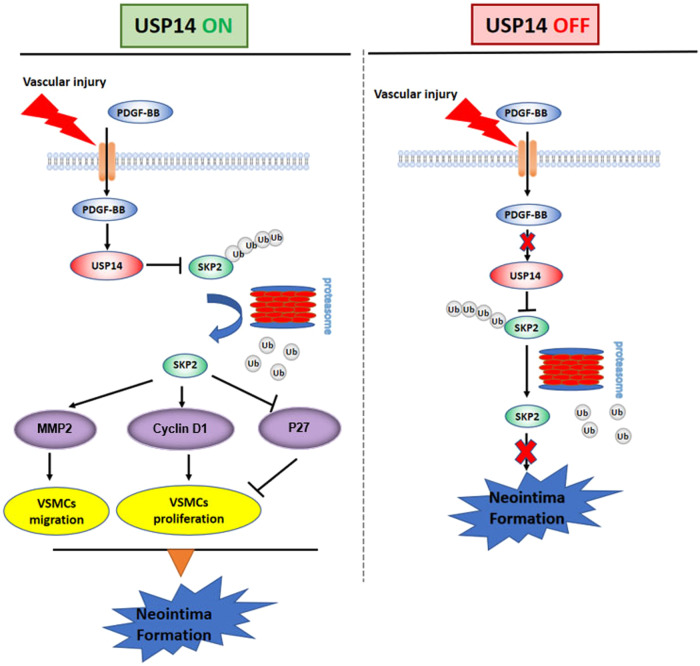
Schematic summary. This study elucidated the function of USP14 in modulating neointima formation. USP14 is localized in SMCs and is upregulated after carotid ligation and PDGF-BB stimuli. USP14 interacts with and stabilizes Skp2 expression by preventing its degradation and subsequently moderating the expression of proliferation-related proteins (Cyclin D1 and p27) and migration protein (MMP2). The inhibition or knockdown of USP14 suppresses VSMC proliferation and decreases neointima formation.

The therapeutically relevant findings of this study are as follows: (i) intimal hyperplasia occurs during arterial restenosis development, atherosclerosis, and vein bypass graft failure [5]; (ii) several studies pointed out the critical role of ubiquitin-proteasome system (UPS) in the initiation and progression of cardiovascular disease through vascular remodeling. The E3 ligases were involved in vascular remodeling via VSMC function; (iii) DUBs control protein’s ubiquitylation in UPS, play essential roles in protein stability, and participate in the progression of many diseases; however, the role of DUBs in neointima formation is yet unknown [20, 21]. Herein, we screened a series of DUBs to observe their protein expression under PDGF-BB stimuli and found that USP14 expression was increased significantly; (iv) USP14 is a DUB primarily reported in cancer development [24, 25]. The present study supported these findings and established a direct role of USP14 in intimal hyperplasia. USP14 inhibition or knockdown reduces cell proliferation in vitro and in vivo. Mechanistically, we identified that USP14 interacts with and stabilizes the Skp2 protein. Moreover, the suppression of USP14 deletion-triggered cell proliferation was rescued by Skp2 overexpression. These findings indicated an essential role of USP14 in vascular remodeling (Fig. 8).

Since the discovery, the DUB family member USP14 has been primarily explored in cancer progression. Recent studies reported that USP14 removes the ubiquitin on androgen receptors in prostate and breast cancer cells to promote cell growth [26, 27]. Conversely, USP14 deficiency regulates the posttranslational modifications of scavenger receptor CD36 to suppress atherosclerosis development [28] and cardiac hypertrophy by regulating GSK-3β phosphorylation [29] and mediates lipopolysaccharide-induced inflammation [30]. Liu et al. reported that USP14 loss inhibits PDGF-BB-induced phenotypic modulation, migration, and proliferation [31]. Next, we postulated that USP14 aggravates arterial injury. In order to explore the level and effect of USP14 in SMCs, we used actin alpha 2, smooth muscle (α-SMA) as a marker to identify the cells. α-SMA is a classic marker expressed in SMCs [32, 33], although it is also detected in non-SMCs under certain conditions [34]. Herein, we found that USP14 is not only expressed in the adventitia and medial layers in the normal artery but also located in the newly formed intima, and it was more than that in the adventitia or medial layers. These results were consistent with those in vitro.

Intima formation was closely associated with USP14 high expression. Thus, we aimed to elucidate the mechanism underlying USP14-promoted intimal hyperplasia. Furthermore, loss- and gain-of-function studies demonstrated a distinct role for USP14 in vascular remodeling. To date, in vitro studies have shown that USP14 deficiency inhibits cell viability using MTS assay. Clone formation and EdU assays verified that USP14 loss induces cell proliferation with or without PDGF-BB treatment. Cell migration and cell cycle were suppressed after USP14 inhibitor treatment in HASMC and A7r5 cells. These results confirmed the function of USP14 in proliferation and migration. Moreover, we used animal models to demonstrate the role of USP14 in intimal hyperplasia. Firstly, we constructed the mice model of carotid ligation. Then, the USP14 inhibitor was applied for treatment. The results showed lesser intimal hyperplasia in the USP14 inhibitor treatment group than in the control group. Additionally, AAV-shRNA USP14-transduced mice exhibited decreased intima areas compared to the AAV-control vector, suggesting that USP14 promotes intimal hyperplasia. These data suggested that USP14 promotes VSMC proliferation to induce intimal hyperplasia with or without PDGF-BB stimuli.

Next, we sought to explore the mechanism of USP14 in HASMC and A7r5 cells. Previous studies have reported that Skp2 promotes cell cycle progression and is a vital factor for VSMC proliferation [14–16]. Herein, we characterized Skp2 as the substrate of USP14 in VSMCs. The present study showed that USP14 interacts with and stabilizes Skp2 protein. Skp2 and its downstream molecule p27 were regulated by USP14 inhibitor or siRNA, resulting in cell cycle arrest from G0/G1 to S phase. Moreover, USP14 loss accelerated the degradation of Skp2 protein by increasing poly- and K48-ubiquitinated Skp2 levels. The overexpression of Skp2 abrogated the cell cycle arrest induced by USP14 deficiency. These findings indicated that USP14 stimulated-intimal hyperplasia was dependent on the Skp2 pathway.

This study has potential limitations. Animal models have more choices. We should choose ones that are closer to vascular injury, such as carotid artery wire injury or femoral artery wire injury. Furthermore, the design of the paper could be further improved. For instance, in the proposal of scientific problems, which could be approached through proteomics, transcriptomics, and so on. However, these shortcomings did not affect our research results. Taken together, this study indicated an essential function of USP14 in the vascular neointima formation and established USP14 as a DUB by removing the ubiquitin molecule on Skp2.

## Materials and methods

### Materials

The drugs, cycloheximide (S7418) and IU1 (S7134), were purchased from Selleckchem (Houston, TX, USA). The following antibodies were obtained from Cell Signaling Technology (Beverly, MA, USA): anti-K48-ubiquitin (#12805), anti-USP14 (#11931), anti-Skp2 (#2652), anti-ubiquitin (#3936), anti-Cyclin D1 (#55506), anti-p27 (#3686), anti-MMP2 (#40994), anti-α-SMA (#19245), anti-Myc-Tag (#2276), anti-UCHL3 (#8141), anti-CUL4A (#2699), anti-USP8 (#11832), anti-STAMBP (#5245), anti-USP13 (#12577), anti-USP7 (#4833), anti-USP15 (#66310), anti-UCHL1 (#13179), anti-CYLD (#8462), and anti-GAPDH (#5174). Anti-USP22 (ab227523) and anti-UCHL5 (ab176377) were procured from Abcam (Boston, MA, USA). Human PDGF-BB was obtained from Peprotech (Cranbury, NJ, USA).

### Cell lines and cell culture

A7r5 cell line was purchased from the Cell Bank of Type Culture Collection of Chinese Academy of Sciences (Shanghai, China), and human aortic smooth muscle cells (HASMC) were obtained from ScienCell (Carlsbad, CA, USA). The cells were cultured in a DMEM medium containing 10% fetal bovine serum (FBS) at 37 °C under 5% CO_2_.

### Cell proliferation analysis

Cell proliferation was detected using cell viability, colony formation, and EdU staining assays, as described previously [35]. First, the cell viability was measured using MTS (Promega, Madison, Wisconsin, USA) reagent. Briefly, cells were seeded in 96-well plates at a density of 40% for 12–24 h. The associated treatment on the adherent cells was carried out for the indicated duration, followed by MTS addition for 3 h. The cell viability was estimated based on absorbance. For colony formation analysis, the treated cells were digested, and the cell number was counted. Number of 4–6 cells per microscopic field of view was seeded into six-well plate and cultured for 10–14 days, followed by fixation with 4% paraformaldehyde and 1% crystal violet staining. Lastly, EdU staining was conducted to evaluate DNA replication. The cells were seeded into chamber slides at a density of 40% and exposed to IU1 or PDGF-BB; the EdU kit was obtained from Ribobio (Guangzhou, China). EdU was added to the cultured cells for 2 h, followed by glycine treatment and permeabilization with 0.5% Triton X-100. Apollo reaction cocktail and 4′,6-diamidino-2-phenylindole (DAPI) from Abcam (Boston, Massachusetts, USA) were used for cell treatment based on the manufacturer’s instructions. The images were acquired using an Olympus microscope.

### Cell cycle analysis

The assay was performed as described previously [36]. Adherent cells were exposed to the inhibitor or siRNA of USP14 with PDGF-BB. Then, the cells were washed with phosphate-buffered saline (PBS), digested with pancreatin, and resuspended in 500 μL PBS. A volume of 2 mL of 70% ethanol was added to PBS, and the cells were incubated at 4 °C overnight, followed by staining with propidium iodide (PI), RNase A, and 0.2% Triton X-100 in the dark. The cell distribution was assessed by flow cytometry.

### Migration assay

We employed scratch and transwell assays to evaluate cell migration. Firstly, a scratch assay was performed, as described below. Cells were seeded into six-well plates at a density of 95% for 12–24 h and treated with the corresponding agents. The cell wound was scraped by a 200-μL pipette tip when the cell density reached 90% confluency. The images of the cell wound were acquired using an inverted Olympus microscope. For transwell assay, the cells were treated, digested, and seeded in the upper chamber at a density of 90% in a serum-free medium. A volume of 600 μL 10% Fetal Bovine Serum (FBS) medium was added to the lower chamber. The migrated cells were collected, fixed, and stained with 1% crystal violet.

### siRNA and plasmid transfection

The assays were performed as described in our previous study [27]. siRNA was purchased from Santa Cruz (Dallas, USA). Cells were seeded into the plates at a density of 60–70% for 24 h and transfected with the mixture containing 500 μL RPMI Opti-MEM, lipofectamine RNAiMAX, and siRNA for 15 min and 6 h. Then, RPMI Opti-MEM was replaced with 10% FBS medium for 48 h. The plasmid was purchased from GeneChem. The mixture containing 500 μL RPMI, Opti-MEM (Gibco), P3000, plasmids, and lipofectamine 3000 was added to the cells.

### Immunofluorescence staining

The assay was conducted as described previously [37]. Cells seeded in the chamber were treated with inhibitor or siRNA of USP14 with PDGF-BB and MYC-Skp2 or MYC-USP14, washed with PBS, fixed, and permeabilized with 0.5% Triton-X for 5 min. Then, the cells were blocked with 5% bovine serum albumin (Sigma) and incubated with the primary antibody in 1% bovine serum album (BSA) overnight, followed by incubation with the secondary antibody (anti-IgG H&L) for 1 h at room temperature. DAPI was used to stain the cell nucleus. Images were captured using a confocal microscope (Leica TCS SP8).

### Real-time quantitative polymerase chain reaction (RT-qPCR)

Total RNA was isolated from the cells using TRIzol reagent (Thermo Fisher, USA). PrimerScript RT Reagent Kit (TaKaRa, Dalian, China) was utilized for reverse transcription of the RNA (1000 ng) into cDNA. Subsequently, RT-qPCR was performed with 1 μg cDNA as the template to detect the mRNA level of the target gene using SYBR Premix Ex Taq^TM^ Kit (TaKaRa).

### Western blot and Co-IP

The assays were conducted as described previously [38, 39]. The cells were seeded into dishes for 24 h, and the proteins were extracted with lysis buffer (CST). The protein concentration was determined using the BCA Kit. 10–20 μL protein lysate about 40–60 μg protein was resolved by SDS-PAGE and transferred to polyvinylidene difluoride (PVDF) membranes. Subsequently, the membrane was blocked with 5% milk-PBST (0.1% PBST) and probed with primary antibody overnight, followed by incubation with secondary antibody for 1 h. The immunoreactive bands were developed using ECL detection reagents, and the membranes were exposed to X-ray films (Kodak, Japan).

The extracted protein was incubated with the mixture containing antibodies and Dynabeads for 1 h. Then, the reaction mixture was washed with PBST and suspended in an SDS loading buffer for Western blot analysis.

### Mouse and complete carotid artery ligation

This assay was performed using C57BL/6 mice (6–8-week-old, male), according to the ARRIVE Guidelines for reporting animal research, and was approved by the Use Committee and institutional animal care at the Animal Center of Guangzhou Medical University. All animals were housed at 22 ± 1 °C, under 65 ± 5% relative humidity, and 12 h/12 h light/dark cycle with free food and water intake. The animals were assigned randomly to three groups: sham (n = 10), control (n = 10), and treated (n = 10). Briefly, the mice were pre-treated with IU1 (20 mg/kg, intraperitoneal injection) or adeno-associated virus (1.09E + 13 PFU/mL was diluted to 1 mL, and 100 μL was injected via tail vein in the mouse). After 3 days, the animals were anesthetized with amobarbital sodium (75 mg/kg, intraperitoneal injection), and the left common carotids proximal to the carotid bifurcation were ligated using 6-0 silk (non-absorbable). The sham group was not ligated. Subsequently, the mice were anesthetized to excise the carotid arteries embedded in paraffin for downstream tests and euthanized by cervical dislocation after the experiment was completed.

### IHC

The tissue was fixed in formalin and embedded in paraffin. The tissue chips were baked at 60 °C for > 2 h, followed by dewaxing and rehydration with xylene and gradient alcohol. After washing with PBS, the tissue chips were incubated with 3% H_2_O_2_ at room temperature for 30 min to block the endogenous peroxidase activity. The antigen retrieval was performed by heating the slides in antigen repair buffer in a microwave oven. Then, the sections were blocked with 5% BSA at room temperature for 20 min and incubated with the primary antibodies overnight at 4 °C, followed by incubation with secondary antibodies at room temperature for 30 min. Subsequently, the sections were developed using avidin DH-biotinylated horseradish peroxidase complex for 30 min (Vectastain ABC Elite Kit, Vector Laboratories) and diaminobenzidine substrate Kit (Vector Laboratories). The sections were counterstained with hematoxylin and sealed with neutral gum before imaging on a digital slide scanner (Leica CS2).

### Statistical analysis

Data are presented as mean ± standard deviation (SD) from three independent experiments. Unpaired Student’s t-test or one-way analysis of variance (ANOVA) was applied to evaluate the statistical probabilities. All statistical analyses were performed using GraphPad Prism8.0 software and SPSS 16.0. P-value < 0.05 indicated statistical significance.

### Supplementary information

Supplementary Data

Original western blot

## Data Availability

Data will be made available upon request.
