# Supplementary material for: USP14 deficiency inhibits neointima formation following vascular injury via degradation of Skp2 protein
Source: Cell Death Discov. 2024 Jun 22;10:295. doi: 10.1038/s41420-024-02069-1 (PMC11193710; doi:10.1038/s41420-024-02069-1)
Supplement: Supplementary file 1 — Supplementary Data [file 41420_2024_2069_MOESM1_ESM.docx]

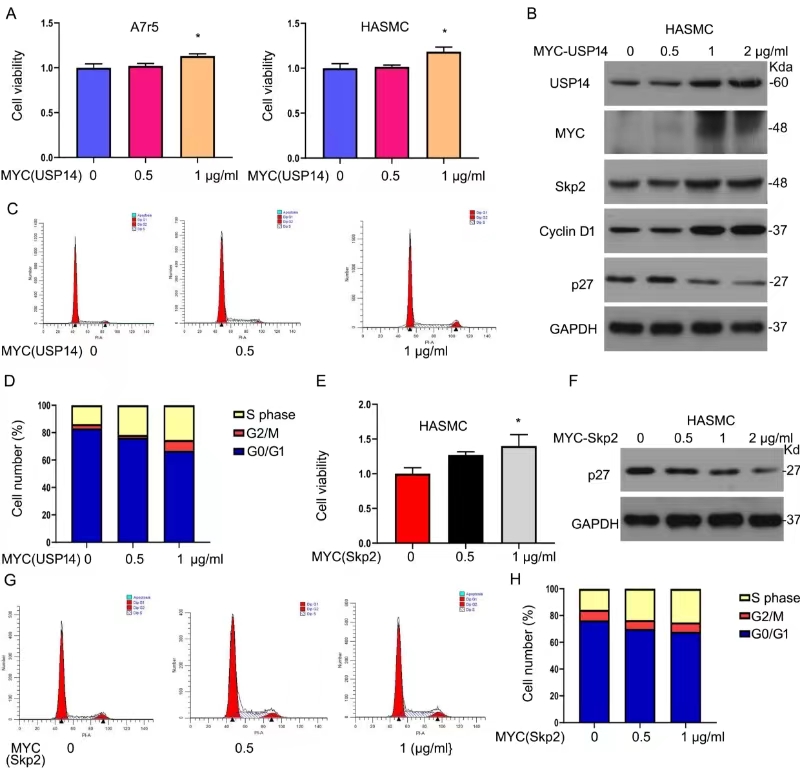


**Fig. S1. The role of USP14 and Skp2 in VSMC proliferation. (A)** MYC-tagged USP14 plasmid was used to transfected into VSMCs for 48 h. MTS assay was performed to evaluate cell viability. **(B)** Transfected cells were subjected to western blot for USP14, MYC, Skp2, Cyclin D1, p27 expression. **(C)** and **(D)** Flow cytometry assay was performed to test cell distribution. **(E)** Cells were transfected with MYC-tagged Skp2 plasmid. MTS assay was performed to detect cell viability. **(F)** Western blot assay was used to test p27 expression. GAPDH was as a loading control. **(G)** and **(H)** Cell cycle was evaluated using flow cytometry. Comparisons were performed by using one way ANOVA.
