# Supplementary material for: USP14 deficiency inhibits neointima formation following vascular injury via degradation of Skp2 protein
Source: Cell Death Discov. 2024 Jun 22;10:295. doi: 10.1038/s41420-024-02069-1 (PMC11193710; doi:10.1038/s41420-024-02069-1)

The original western blots were listed as following.

Fig 1A

UCHL3

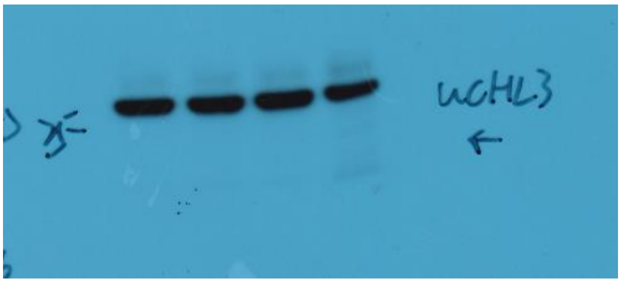

USP22

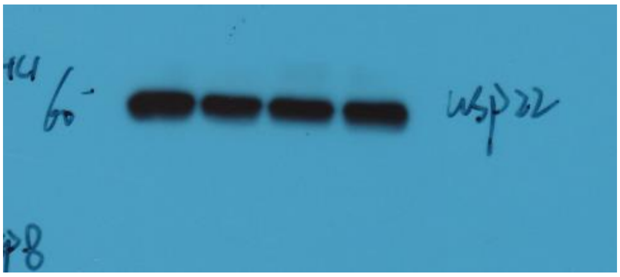

CUL4A

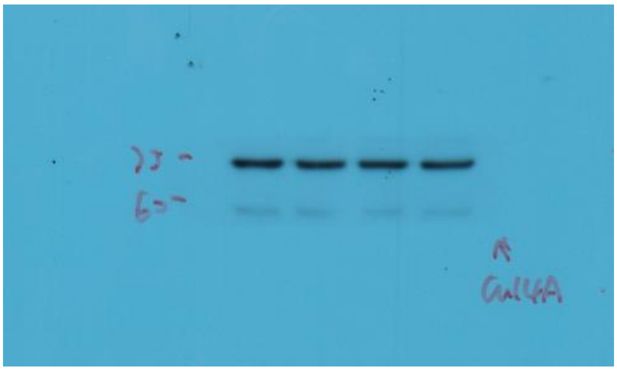

USP14

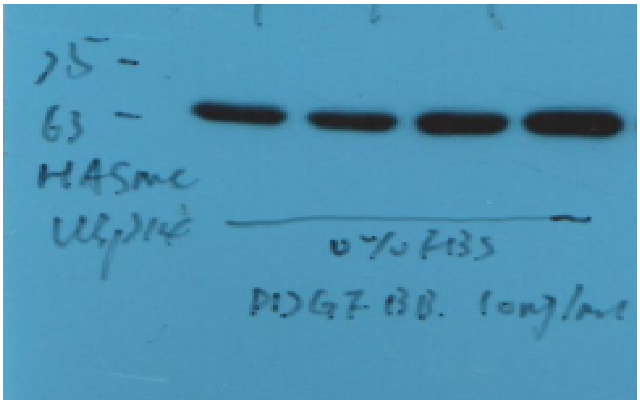

USP8

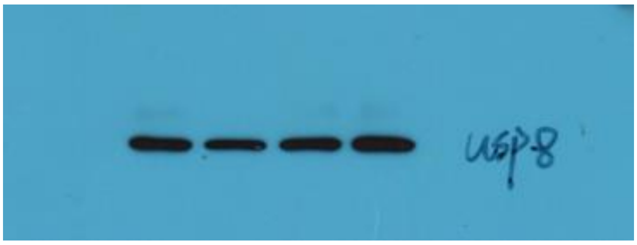

STAMBP

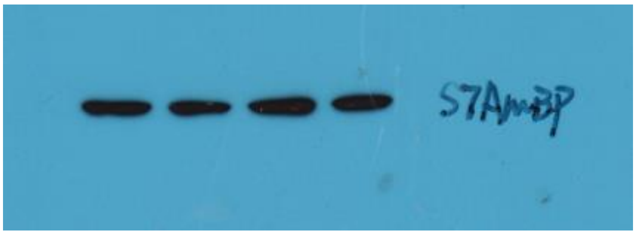

USP13

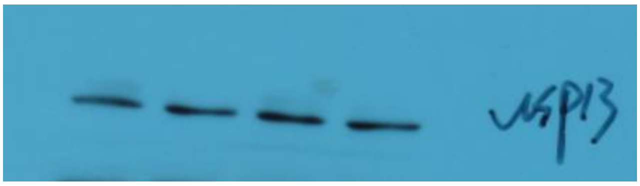

USP7

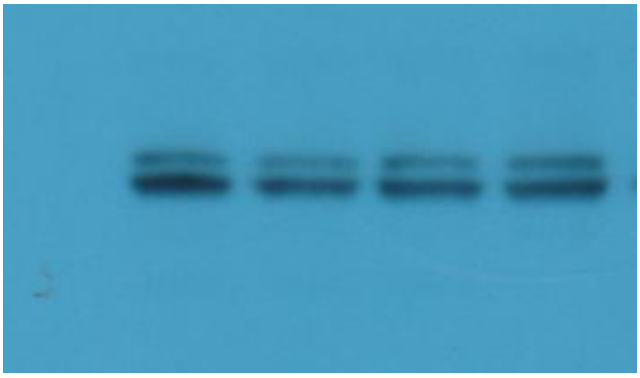

USP15

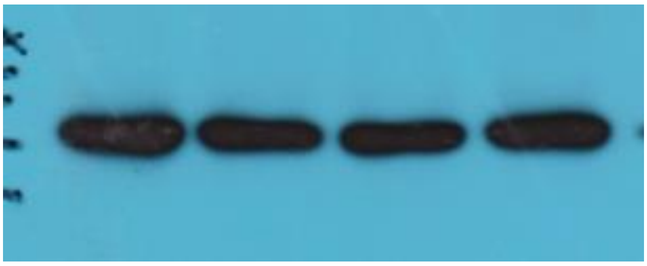

UCHL5

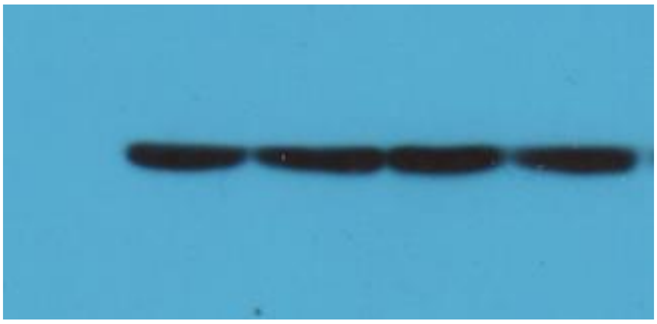

UCHL1

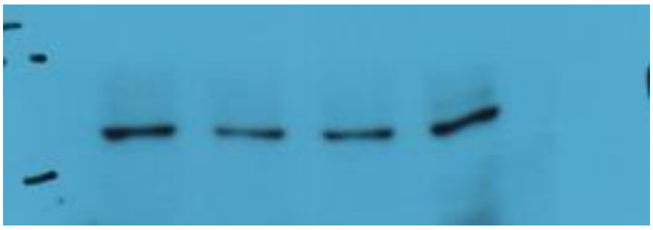

CYLD

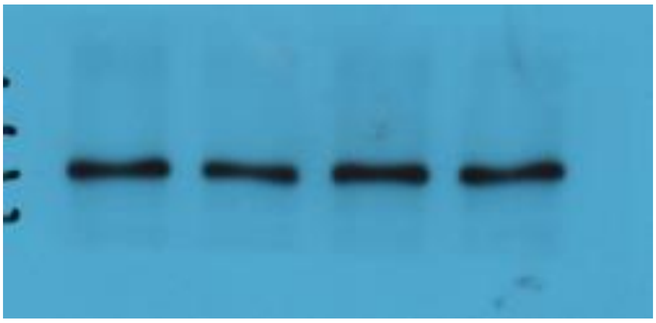

GAPDH

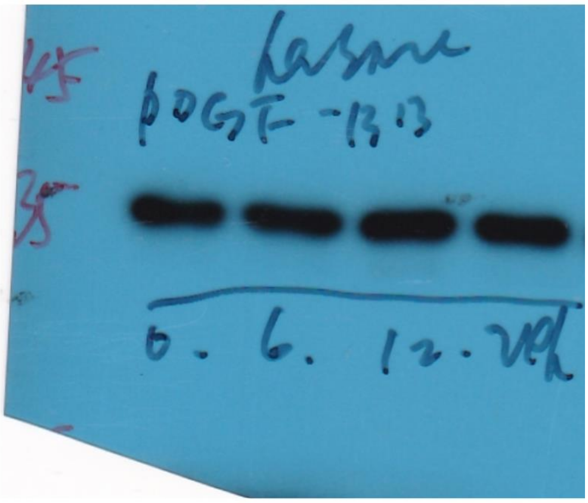

Fig 1B

HASMC
Cyclin D1

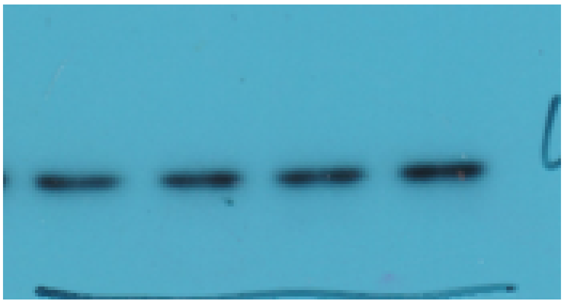

GAPDH

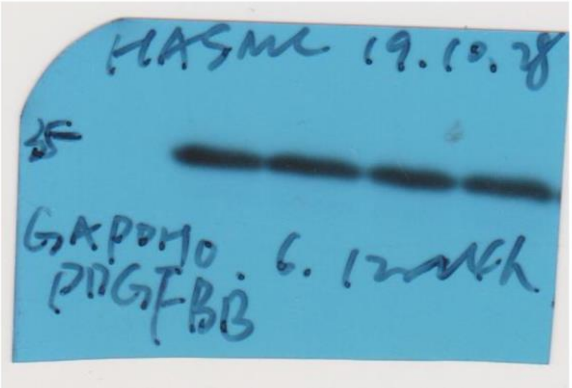

A7r5
Cyclin D1

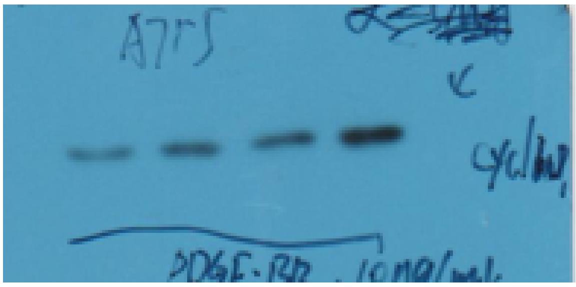

GAPDH

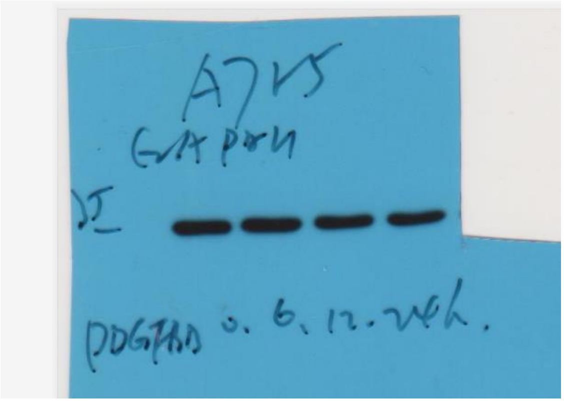

Fig 3E

HASMC
Cyclin D1

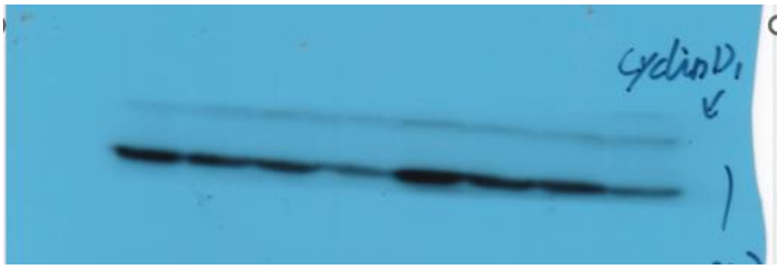

GAPDH

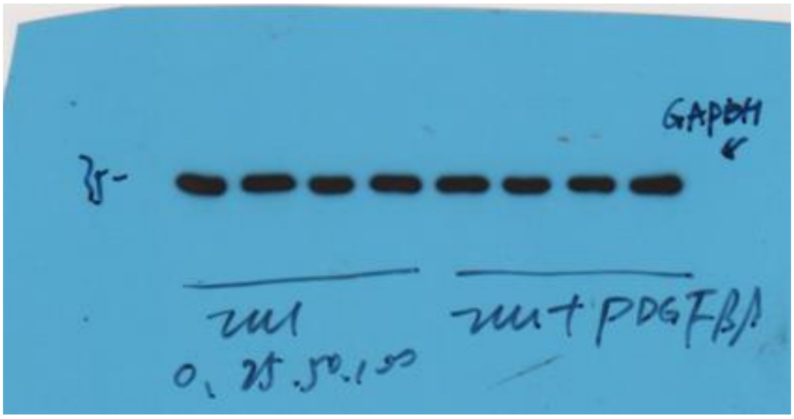

A7r5
Cyclin D1

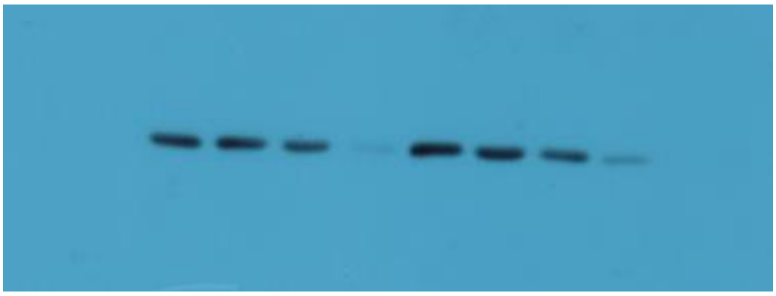

GADPH

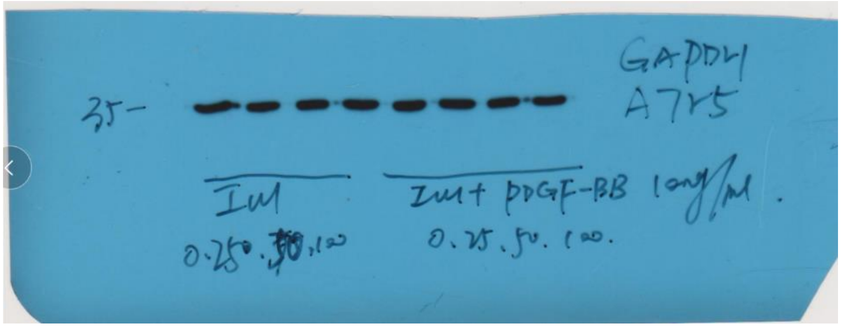

Fig 3F

Cyclin D1

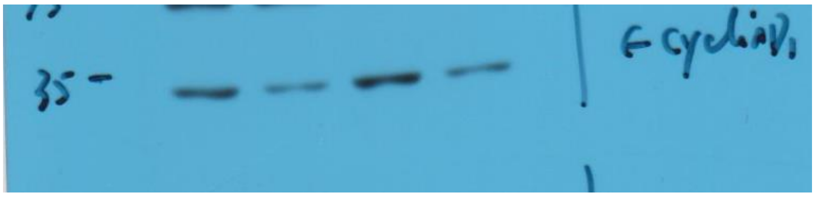

GAPDH

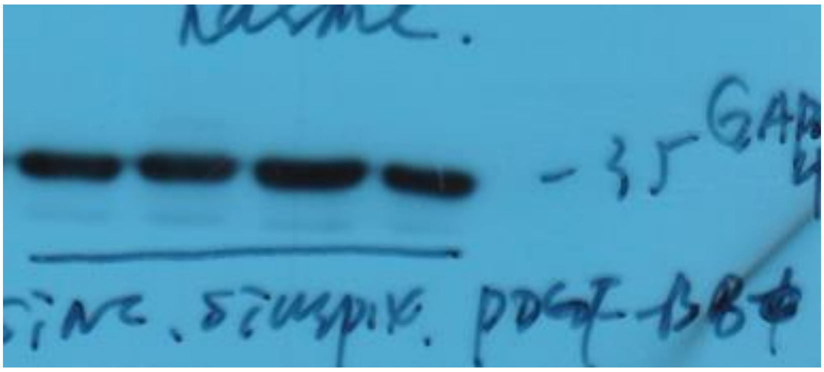

Fig 3I

MMP2

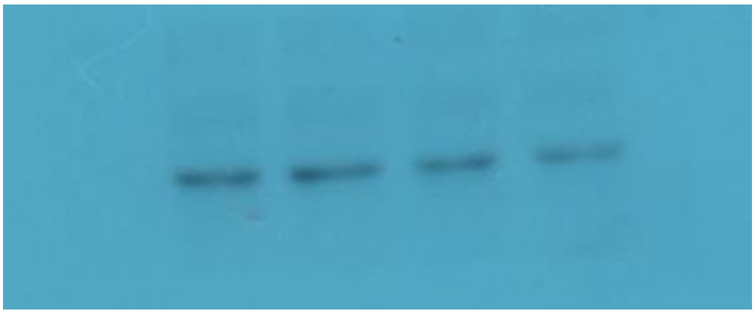

GAPDH

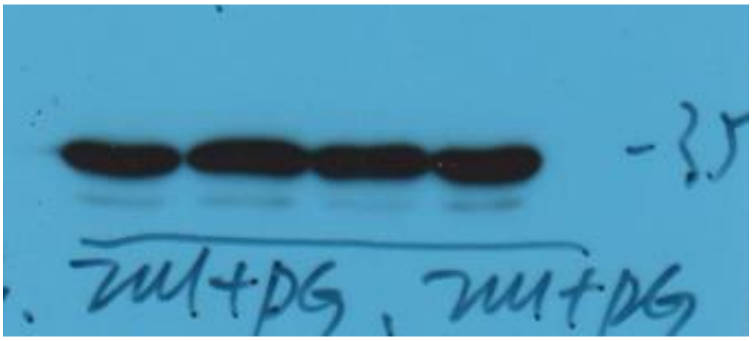

Fig 4A

HASMC
Skp2

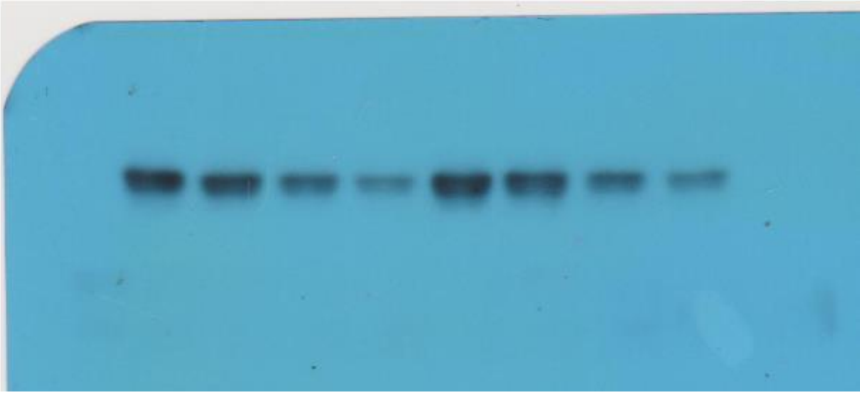

GAPDH

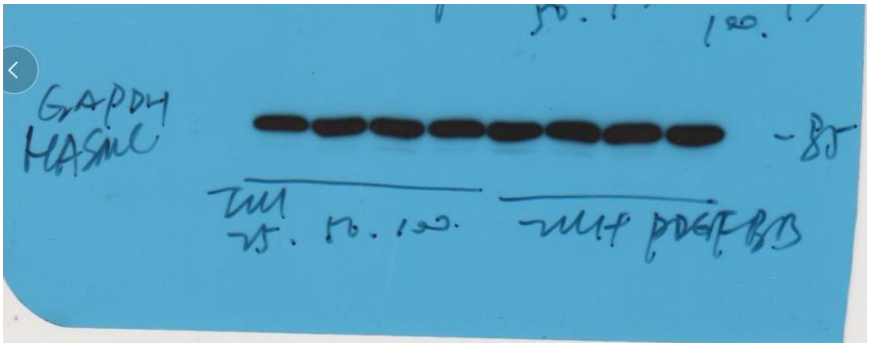

A7r5
Skp2

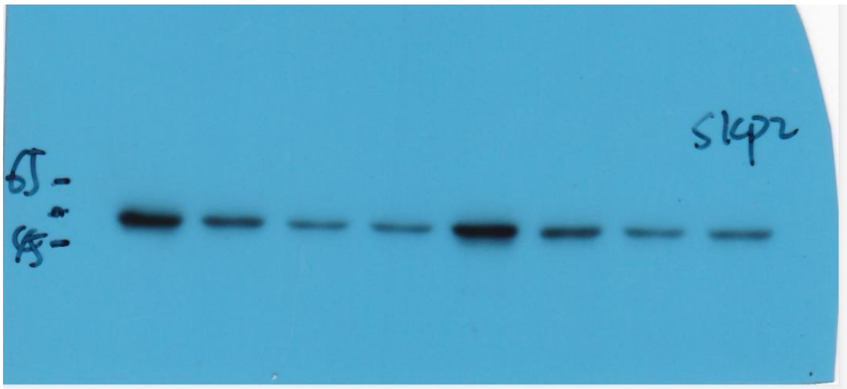

GAPDH

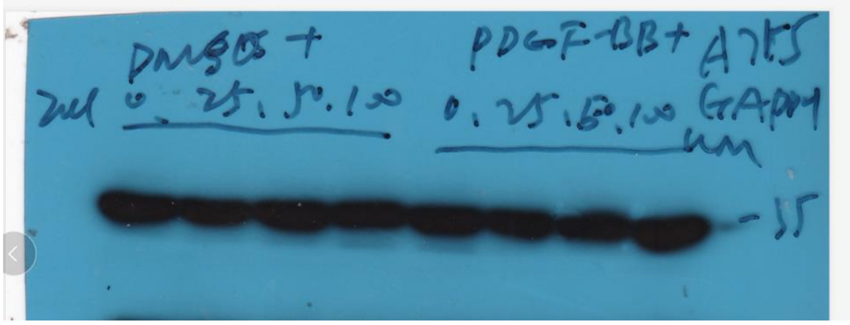

Fig 4B

Skp2

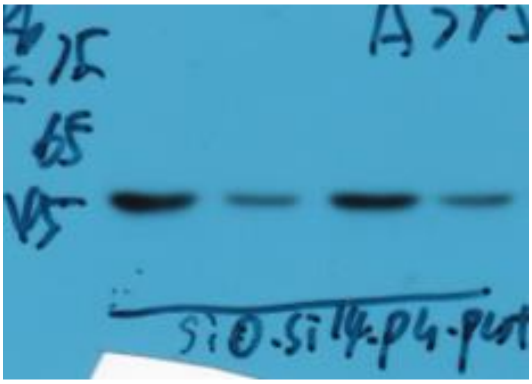

GAPDH

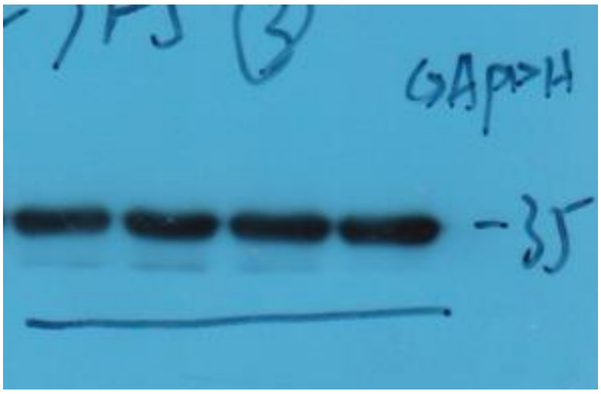

Fig 4C
Skp2

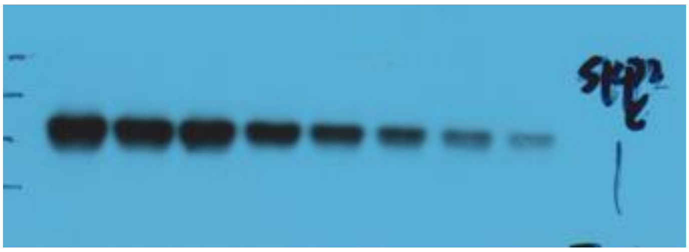

GAPDH

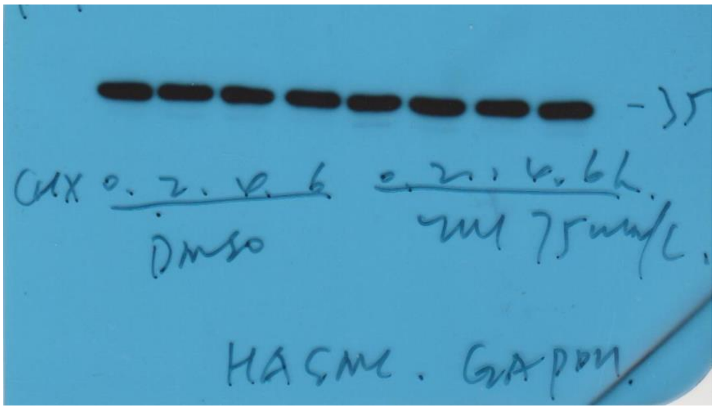

Fig 4D

USP14

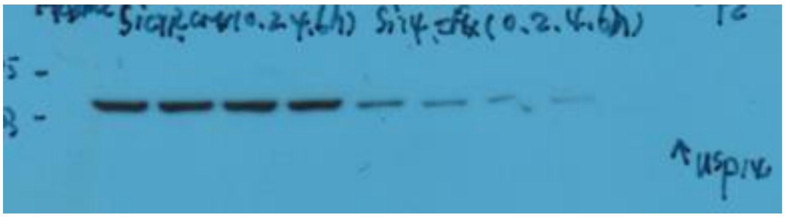

Skp2

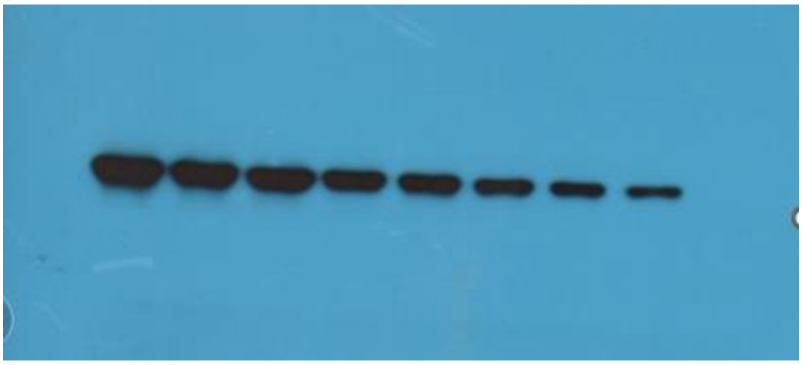

GAPDH

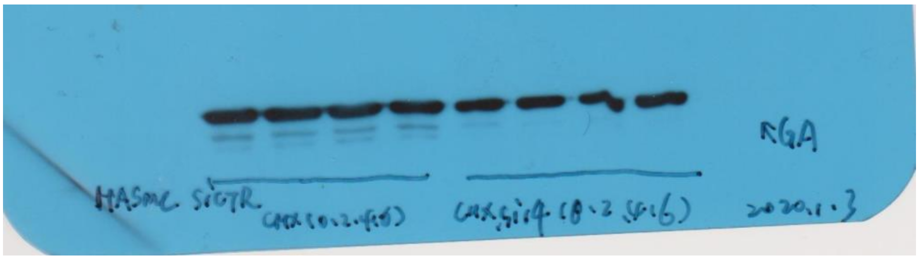

Fig 4E
MYC

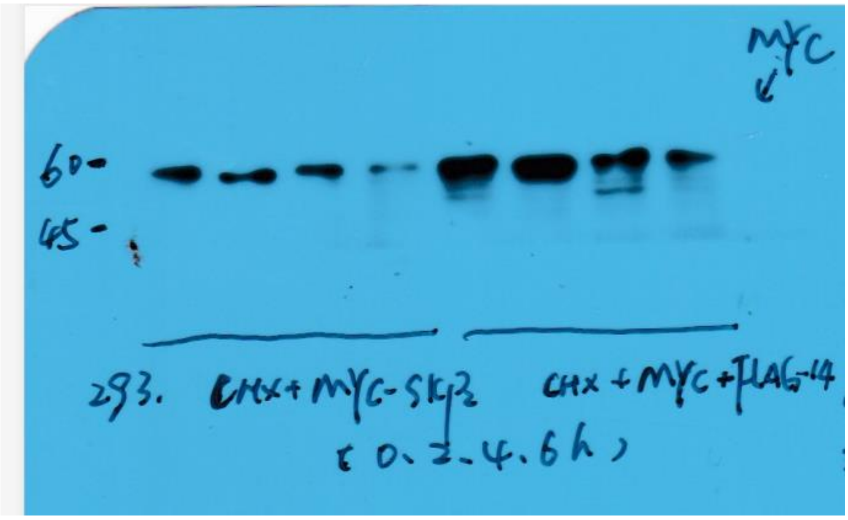

GAPDH

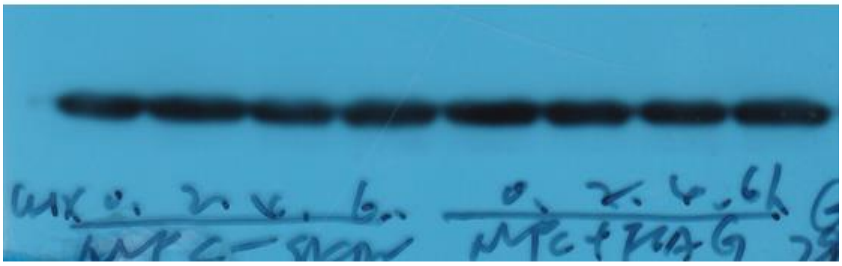

Fig 5A

IP: Skp2
USP14

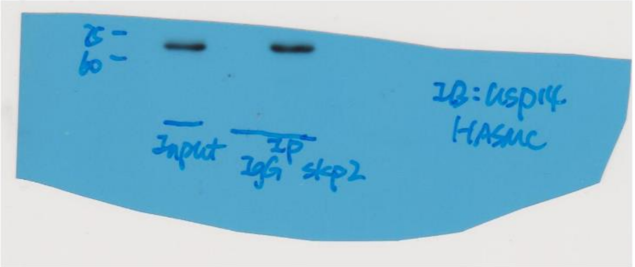

Skp2

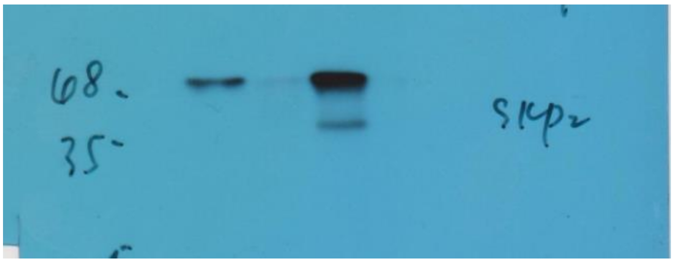

IP: USP14
USP14

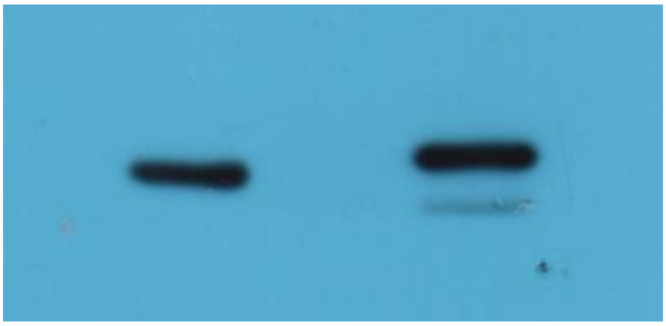

Skp2

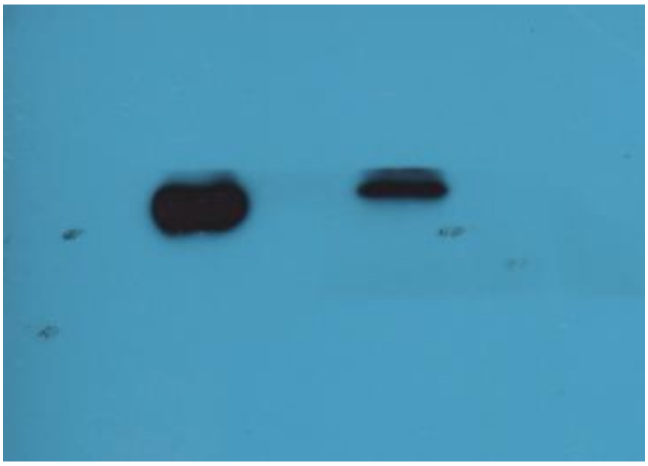

Fig 5B

Input His

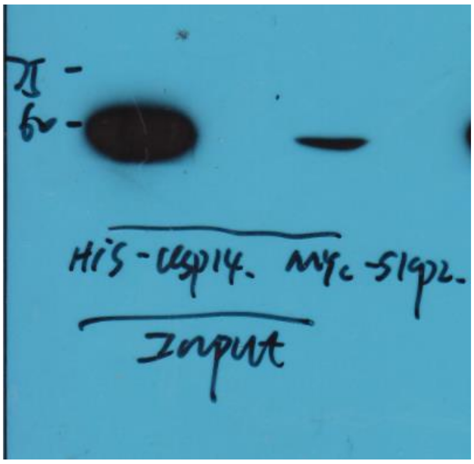

Input Myc

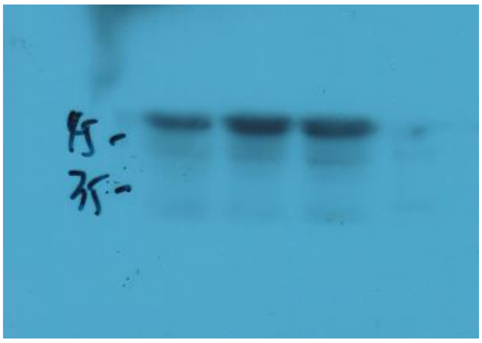

IP His

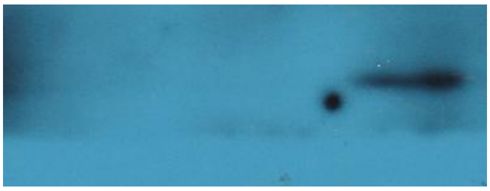

IP Myc

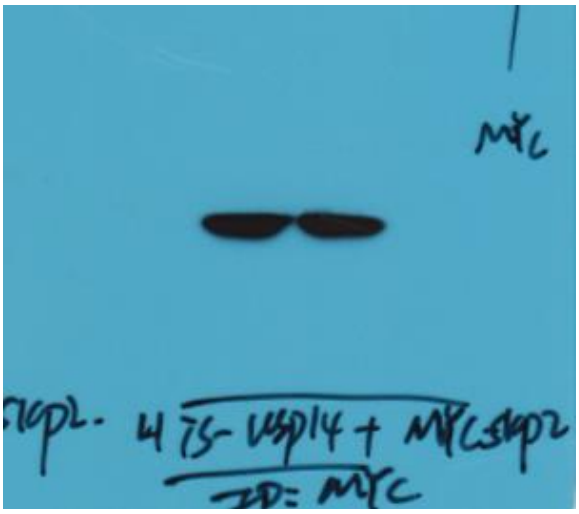

Fig 5D

Input Ub

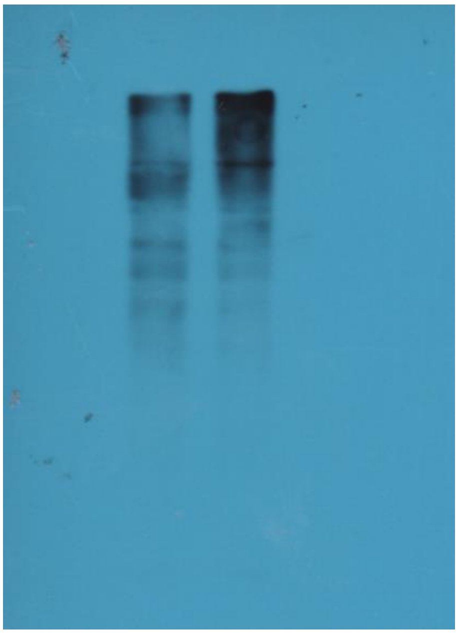

Input K48

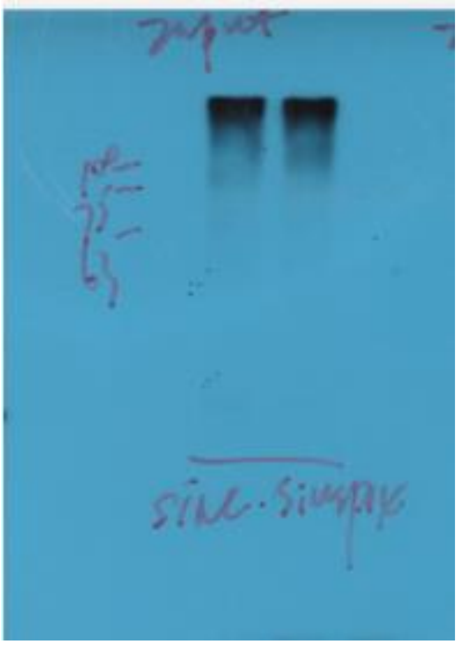

Input Skp2

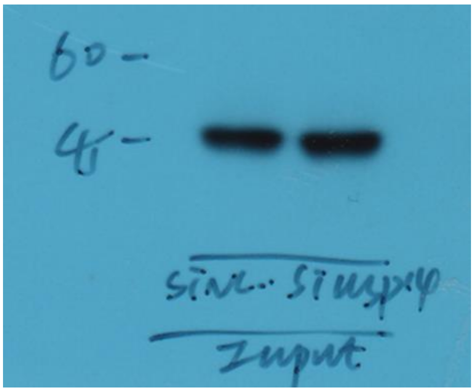

input
GAPDH

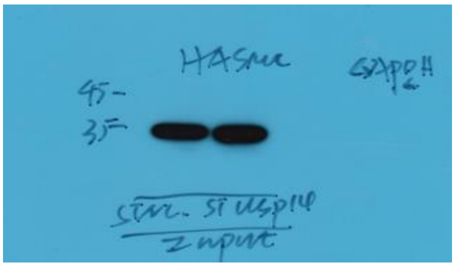

IP Ub

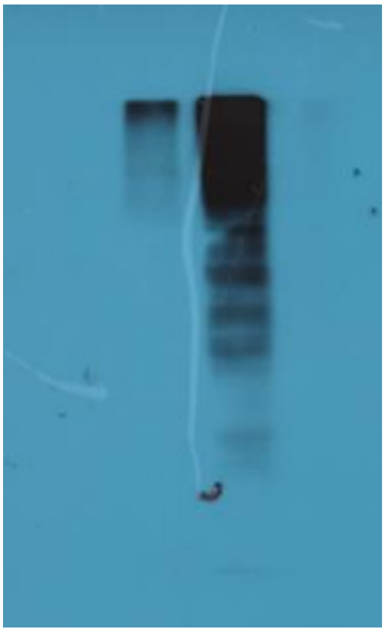

IP K48

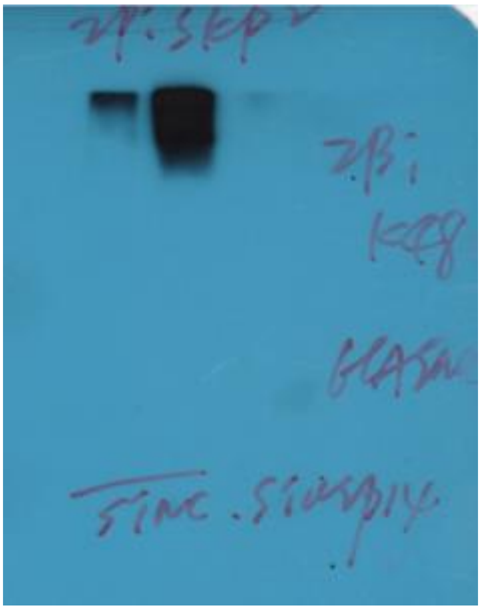

IP Skp2

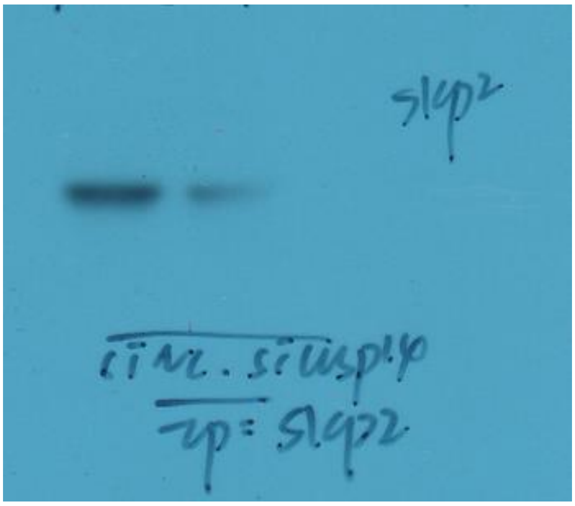

Fig 5E

Input K48

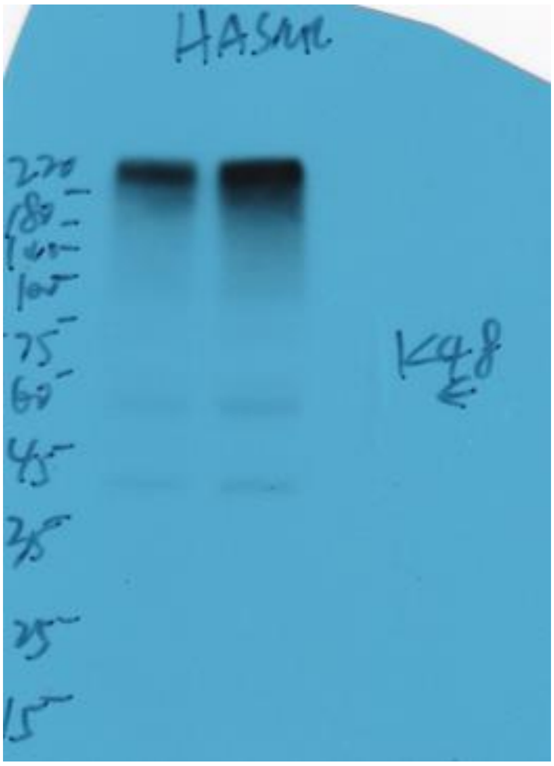

Input Skp2

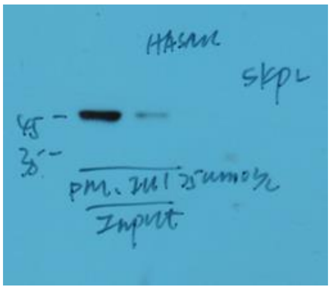

GAPDH

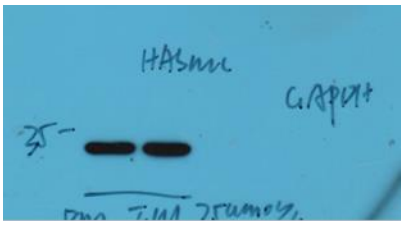

IP K48

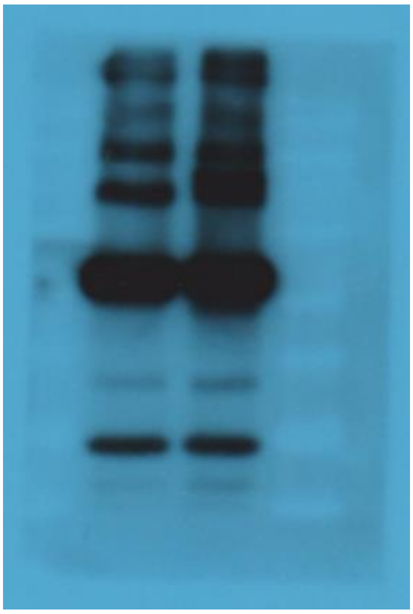

IP Skp2

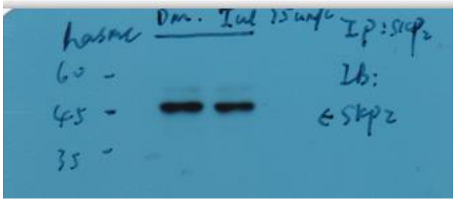

Fig 5F

Input HA

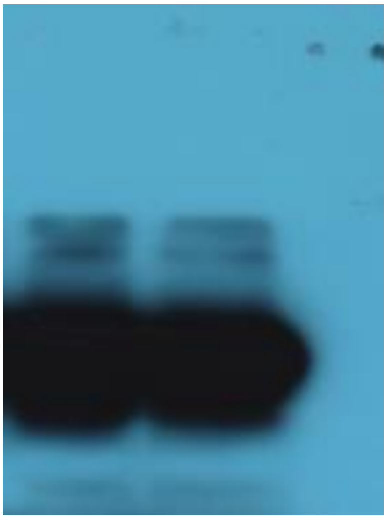

Input MYC

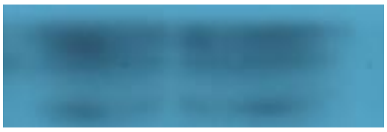

Input His

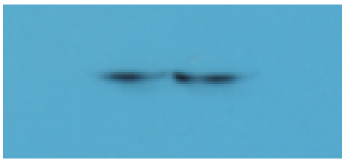

Input GAPDH

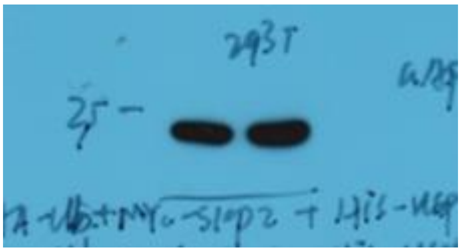

IP HA

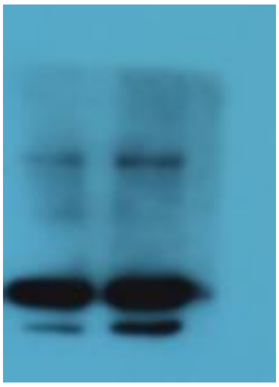

IP MYC

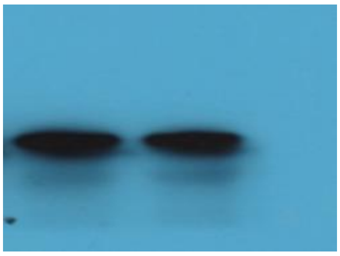

Fig 6D

Skp2

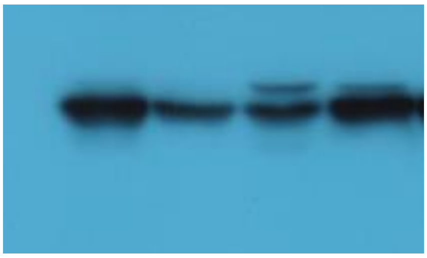

P27

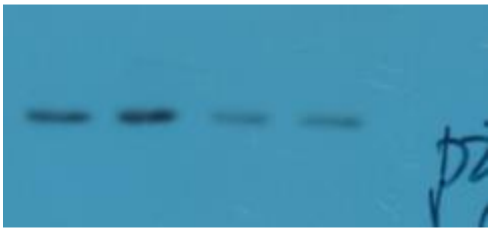

USP14

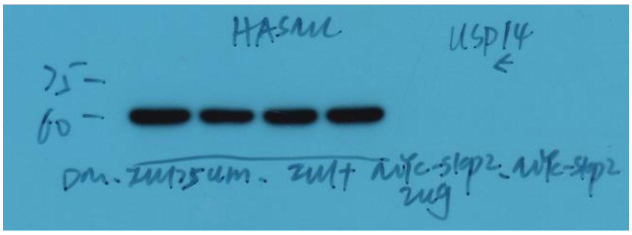

GAPDH

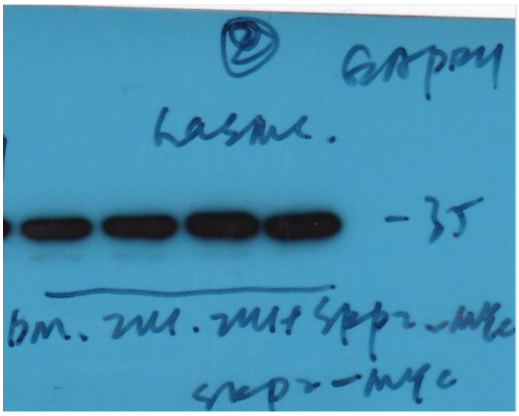

Fig 6E

Skp2

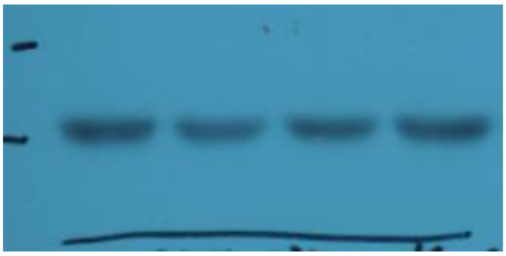

P27

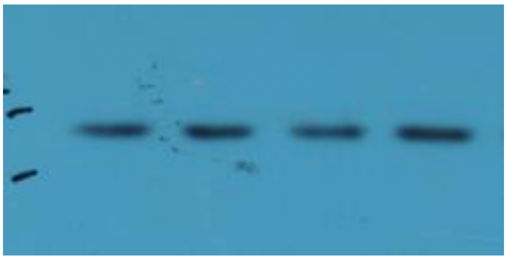

USP14

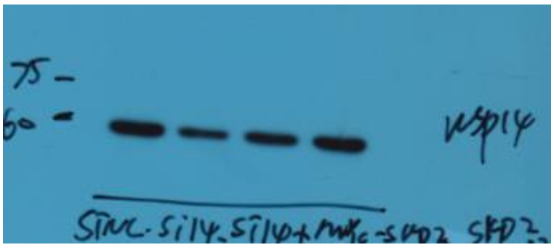

GAPDH

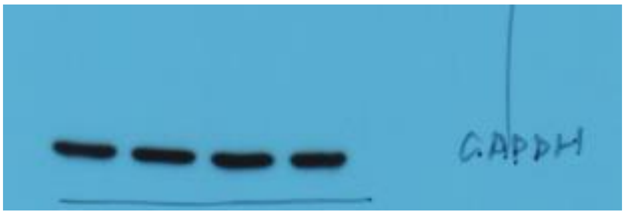

Fig S1 B

USP14

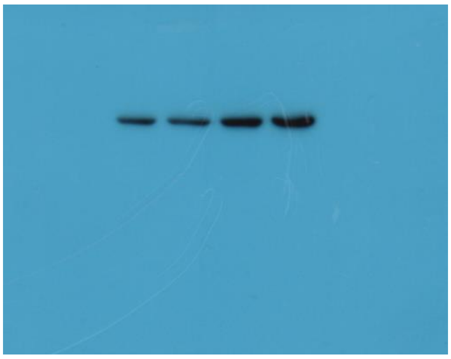

MYC

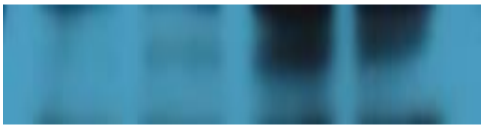

Skp2

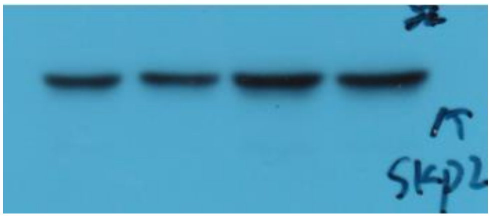

Cyclin D1

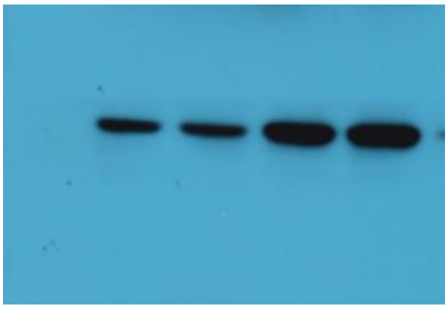

P27

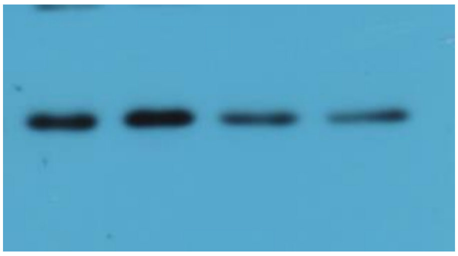

GAPDH

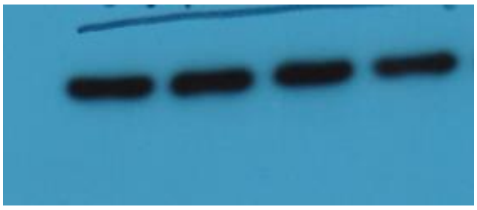

Fig S1 F

P27

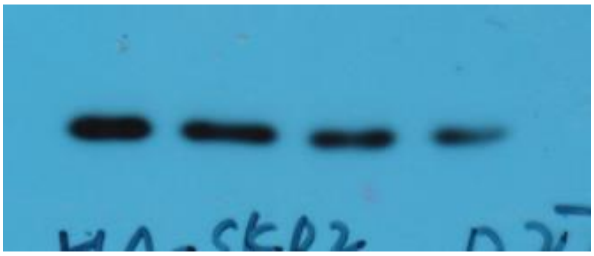

GAPDH

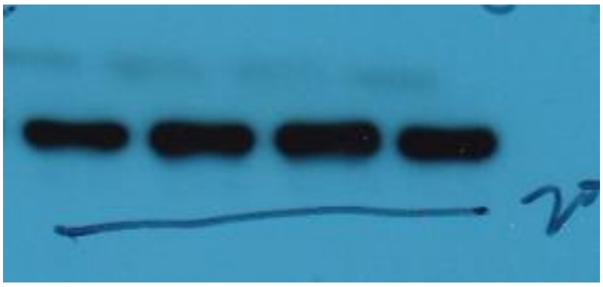

Supplement: Supplementary file 2 — Original western blot [file 41420_2024_2069_MOESM2_ESM.pdf]
